# Associations between socioeconomic status and physical activity among older adults: cross-sectional results from the OUTDOOR ACTIVE study

**DOI:** 10.1186/s12877-022-03075-7

**Published:** 2022-05-06

**Authors:** Imke Stalling, Birte Marie Albrecht, Linda Foettinger, Carina Recke, Karin Bammann

**Affiliations:** Institute for Public Health and Nursing Research (IPP), University of Bremen, Grazer Straße 2a, 28359 Bremen, Germany

**Keywords:** Physical activity, Socioeconomic status, Social class, Older adults, Elderly, Accelerometer

## Abstract

**Background:**

Regular physical activity (PA) is an important strategy for healthy ageing. Socioeconomic status was found to be a key determinant of PA, however, evidence on associations between socioeconomic status and PA among older adults is limited. The aim of this study was to contribute to research on the associations of socioeconomic status and PA among older adults by including self-reported and objectively measured PA data. Furthermore, we examined the self-reported PA data more closely by looking at the activities separately.

**Methods:**

Cross-sectional data of 1507 participants (52.5% female) of the OUTDOOR ACTIVE study between 65 and 75 years, residing in Bremen, Germany, were included in the analyses. Self-reported PA was assessed via questionnaire and comprised all organised and non-organised activities. For analyses, mean hours per week of total and moderate to vigorous PA, and mean metabolic equivalents per week were used. Objectively measured PA was assessed using accelerometers over seven consecutive days. Socioeconomic status was included as an additive social class index containing education, income, and occupation. To test for associations between PA and socioeconomic status, linear regressions were carried out.

**Results:**

Self-reported PA showed significant negative associations with socioeconomic status for both men and women. Objectively measured PA was positively associated with socioeconomic status, which was significant in men but not in women. When examining physical activities separately, time spent on housework, gardening, biking, and walking decreased with increasing socioeconomic status. Women in the second SES quintile and men in the third quintile reported the most, and women in the first quintile and men in the fifth quintile the least hours per week spent on exercise.

**Conclusions:**

The results of this study contributed to the existing research gap on the associations of socioeconomic status and PA among older adults. Moreover, we provided information on both self-reported and objectively measured PA, and showed the discrepancies in the two methods’ results. These findings can help to develop PA promotion interventions targeting specific socioeconomic status groups and to develop accurate, valid, and reliable self-reported and objective measurements of PA for older adults.

**Supplementary Information:**

The online version contains supplementary material available at 10.1186/s12877-022-03075-7.

## Background

The progressing demographic change and the associated increasing proportion of older adults present the society with new challenges regarding healthy ageing [1]. One important strategy is regular physical activity (PA) [2], which improves quality of life, and lowers the risk of numerous non-communicable diseases, disabilities and mortality [3, 4]. Adults are recommended to engage in moderate PA of at least 150 min per week, according to the World Health Organization (WHO) [4]. However, the prevalence of people reaching this amount of PA decreases with age [5], with 18.0% of the 60- to 69-year-olds and only 13.6% of the 70- to 79-year-olds in Germany [6].

Several determinants of PA among older adults have been identified in past research, such as age, gender, self-reported health, and environmental factors [7, 8]. While this line of research also suggests socioeconomic status (SES) as a key factor of PA (9–11), the evidence on associations between SES and PA among older adults is limited [9, 10]. Knowledge on the possible associations between SES and PA is, however, important, since participation and compliance in health promotion interventions are highly influenced by socioeconomic factors [11, 12]. The existing evidence shows contradictory findings, such as a systematic review by Koeneman et al. [13] found out. While several studies suggest that higher SES is associated with lower levels of physical inactivity as well as higher levels of leisure-time PA (LTPA), and overall PA among older adults [9, 10, 14], others found opposite results [13, 15]. Several non-significant associations and negative associations between PA and education, income, and employment status were found in a systematic review [13]. Similar results were reported by Moschny et al. [15], who found no associations between education and time spent on sporting activities for women and men, and negative associations for older men regarding time spent on domestic activities. One possible explanation for these contradictory findings is the inconsistent assessment of PA and that most studies use self-administered questionnaires for measuring PA [8–10, 13–15]. Additionally, a unified approach to measure SES is missing [16], which leads to research often including single socioeconomic factors instead of SES [10, 17–19].

Addressing this research gap, the aim of this study is to contribute to research on the associations of SES and PA among older adults, using an additive social class index comprising income, education, and occupation. We examine the self-reported PA data more closely by looking at the activities separately. Furthermore, we strengthen the evidence on PA by also using objectively measured accelerometer data to investigate if any differences exist.

## Methods

### Study design and sample

This cross-sectional analysis stems from the OUTDOOR ACTIVE study, which is a subproject of AEQUIPA (Physical activity and health equity: primary prevention for healthy ageing), a prevention network in north-western Germany [20]. The main research goals of OUTDOOR ACTIVE focus on assessing PA in older adults and investigating barriers and drivers for being physically active. Furthermore, a community-based outdoor PA promotion program is being developed using participatory methods and is subsequently implemented [21]. The study consists of two parts: a pilot study (February 2015 to January 2018) and a cluster-randomized controlled trial (c-RCT; February 2018 to March 2022). In both parts, a baseline and follow-up survey were carried out. They comprised a short physical examination, followed by a fitness test [22], as well as seven-day accelerometry to objectively measure PA. Furthermore, participants were given a self-administered paper-pencil questionnaire including intrapersonal, interpersonal, and environmental determinants of PA [21, 23].

Eligible for participation were all inhabitants of defined subdistricts of Bremen (pilot study: Arbergen, Hastedt, Hemelingen, Mahndorf, Sebaldsbrueck; c-RCT: Blumenthal, Burg-Grambke, Gete, Lehe, Lehesterdeich, Neustadt, Ohlenhof, Ostertor), who were between 65 and 75 years old, and not institutionalised. Address data were provided by the registry office in Bremen. All potential participants were initially contacted by post and later by phone. In total, 11,079 individuals were eligible for study participation. Of these, 1113 people were excluded (acute health problems *n* = 461; language barriers *n* = 77; moving out of the study region *n* = 450; deceased *n* = 125). A further 3425 individuals were never reached and 4247 refused participation. One hundred fifty-one persons of the subdistrict Lehesterdeich were never contacted, since the survey period for that region ended and the sample size had already been exceeded at that point. Effectively, 2143 participated in at least one part of either the pilot study or the c-RCT, of which the 1507 participants, who partook in the accelerometer measurement, were included in the present study.

All participants provided written informed consent and both study parts were approved by the ethics committee of the University of Bremen.

### Measures

#### Accelerometer-assessed physical activity

PA was measured objectively using the ActiGraph GT3x-BTw *(*ActiGraph LLC, Pensacola, FL, USA) accelerometers. These devices measure accelerations and decelerations of the body in three axes [24]. Sampling frequency was set to 30 Hz, count data were downloaded with ActiLife *(*Version 6.13.3 ActiGraph LLC, Pensacola, FL, USA), and prepared for statistical analyses. Participants were asked to wear them on their non-dominant wrist for 7 days consecutively, ideally for 24 h straight. Counts, which are provided by the ActiLife software, are unitless calibrated and band-pass filtered accelerometer data [25]. Vector magnitudes were calculated from the count data of the three axes and integrated to 1 minute [26]. Non-wear time was defined as 90 consecutive minutes with zero counts [27]. One day runs from 0:00 h to 24:00 h and from the maximum of 1440 min per day, average daily counts per minute (CPM, calculated from the vector magnitudes) were included in the analyses. Average daily CPM reflect the total amount of PA. The use of this measure is validated against the doubly labelled water method [28].

#### Self-reported physical activity

Self-reported PA was assessed using the baseline self-administered questionnaire. Participants reported all currently performed organised (e.g., sports club, sports group, or gym) as well as non-organised activities, including household chores or gardening, and stated the hours per week for the individual activities (free-text).

PA levels for each activity were categorized using the metabolic equivalents (METs) according to the Compendium of physical activity by Ainsworth et al. [29], with moderate to vigorous PA (MVPA) being ≥3 METs.

#### Sociodemographic information

Information on age, sex, and marital status, were assessed using the self-administered questionnaire. Self-reported health status was assessed using a question from the SF-36 [30].

Each participant was assigned a SES by calculating an additive social class index based on Helmert et al. [31],and Winkler and Stolzenberg [32] including education, income, and occupation. For education, self-reported data on school qualification and professional degree were assessed using adapted questions from the German health interview and examination survey for adults (DEGS) [33]. Based on the answers school years and training years were calculated and summed up to educational years as educational status. Net household income was also assessed using an adapted question from DEGS [33], with categories ranging from “less than 500€” to “more than 4000€”. For occupation, the participants were asked for their occupational history by stating each occupation they have ever carried out and the years. For the SES additive index, the occupation that was carried out the longest was classified by the Standard International Occupational Prestige Scale based on Helmert et al. [31]. To calculate the additive index, the variables were scaled to percent, with possible values from 0 to 100%. Missing values were imputed in SPSS 22 (IBM Corp. Armonk, NY) by aggregating five iterations of multiple imputation (method of chained equations imputation using linear regressions [34]) into their mean values. The three components education, income, and occupation were summed up (using equal weights) to the SES index and divided by three. The SES index was categorized into quintiles [23], with the first quintile representing the lowest SES category and the fifth quintile the highest SES category. The number of missing values can be found in Additional file 1.

### Statistical analyses

Descriptive analyses contain absolute and relative frequencies for marital status, SES, occupational status, and self-reported health status. Means and standard deviations were calculated for age, self-reported PA (hours per week of total PA, hours per week spent in MVPA, METs per week), total objective PA (CPM), as well as self-reported time spent on physical activities. The activities were categorized into housework, gardening, biking, walking, and exercise to reflect the most important daily domains of PA.

To test for associations between PA and SES, linear regressions were carried out with self-reported PA variables and objectively measured PA as dependent variables. SES was included as a continuous variable. Analyses were adjusted for age and self-reported health and unstandardized coefficient B is being reported. Kruskal-Wallis-Tests were carried out to test for significant differences between the SES quintiles; *p*-values are being reported. All analyses were done separately for women and men, and analyses regarding PA were additionally stratified by SES.

All statistical analyses were conducted with SPSS 22.0 (IBM Corp. Armonk, NY).

## Results

The characteristics of the study population (*n* = 1507) are displayed in Table 1. 52.5% of the participants were female and the majority was married, with 56.3% of women and 80.1% of men. The majority (60.7%) of women and men pertained to the second, third, or fourth SES quintile. 21.0% of women and 12.8% of men belong to the first SES quintile, whereas 18.3% of women and 26.5% of men have a higher SES. 13.7% of women and 18.2% of men have a paid occupation. Most participants (women: 83.3%; men: 87.2%) reported their health status as at least good. The mean age of the study population was 69.5 ± 2.8 years.

**Table 1:** Characteristics of the study population

	Women (*n* = 791)	Men (*n* = 716)
	Mean (SD)	Mean (SD)
Age (years)	69.6 (2.9)	69.5 (2.8)
	n (%)	n (%)
Marital status		
Married	427 (56.3)	546 (80.1)
Divorced	142 (18.7)	69 (10.1)
Widowed	124 (16.3)	26 (3.6)
Unwed/single	66 (8.7)	41 (6.0)
Self-reported health status		
Less good or bad	127 (16.7)	88 (12.8)
Good	443 (58.3)	392 (57.1)
Very good or excellent	190 (25.0)	207 (30.1)
Occupational status		
Paid occupation	108 (13.7)	130 (18.2)
No occupation	683 (86.3)	586 (81.8)
Socioeconomic status		
1st SES quintile (lowest)	161 (21.0)	88 (12.8)
2nd SES quintile	173 (22.6)	107 (15.5)
3rd SES quintile	150 (19.6)	145 (21.0)
4th SES quintile	142 (18.5)	167 (24.2)
5th SES quintile (highest)	140 (18.3)	183 (26.5)

*SD* Standard deviation

Table 2 shows the mean self-reported and objectively assessed PA indicators stratified by sex and SES. Regardless of sex, the self-reported mean hours per week of total PA and MVPA, as well as the mean METs per week decrease with increasing SES. In the lowest quintile, women stated the highest amount of PA (13.27 ± 14.49 h/week) and MVPA (6.61 ± 7.36 h/week), whereas women in the fifth SES quintile reported the lowest (4.76 ± 6.73 h/week of PA and 3.14 ± 4.26 h/week of MVPA). This can also be observed for METs/week, as women with a lower SES reported activities with the highest amount of METs (49.63 ± 53.81 METs/week) and women with a higher SES the lowest amount (20.88 ± 28.12 METs/week). Similar results are seen among the men, with the most hours per week of PA and MVPA in the lowest quintile (13.73 ± 15.82 h/week and 9.23 ± 11.55 h/week, respectively), and the lowest time in the highest quintile (5.44 ± 7.23 h/week of PA; 4.39 ± 5.52 h/week of MVPA). With regards to the PA levels of the self-reported activities, men in the first SES quintile stated the highest amount of METs per week (56.71 ± 66.29) and in the fifth quintile the lowest (25.56 ± 32.31 METs/week). This observed pattern is not applying to objectively measured PA. The highest mean CPM among women, however, were seen among the third quintile (1885.91 ± 636.76 CPM) and the lowest among women in first quintile (1801.83 ± 472.15 CPM). Men in the second SES quintile showed the lowest CPM (1449.01 ± 433.45 CPM) and in the fifth quintile the highest (1624.98 ± 435.61 CPM).

**Table 2:** Physical activity indicators by socioeconomic status stratified by sex; descriptive statistics and linear regression

	1st SES quintile (*n* = 164)	2nd SES quintile (*n* = 176)	3rd SES quintile (*n* = 150)	4th SES quintile (*n* = 142)	5th SES quintile (*n* = 141)	Linear regression B for SES percent (95% CL)		Kruskal-Wallis-Test
	Mean (SD)					adj. For age	adj. For age and health status	*p*-value
Women								
Questionnaire								
PA, hrs/week	13.27 (14.49)	12.17 (13.83)	9.82 (12.19)	7.99 (12.23)	4.76 (6.73)	**−0.22 (−0.29, −0.15)**	**−0,22 (−0.29, − 0.17)**	**< 0.01**
MVPA, hrs/week	6.61 (7.36)	6.14 (7.33)	5.55 (6.53)	4.87 (7.24)	3.14 (4.26)	**−0.09 (− 0.12, − 0.05)**	**−0.09 (− 0.13, − 0.05)**	**< 0.01**
METs/week	49.63 (53.81)	47.22 (53.26)	39.21 (46.91)	31.92 (47.05)	20.88 (28.12)	**−0.76 (−1.02, − 0.50)**	**−0.76 (− 1.04, − 0.49)**	**< 0.01**
Accelerometer								
Total PA (CPM)	1801.83 (472.15)	1845.52 (459.66)	1885.91 (636.76)	1814.07 (458.57)	1823.30 (457.93)	0.39 (−2.38, 3.17)	−0.72 (−3.56, 2.12)	0.89
	1st SES quintile (*n* = 88)	2nd SES quintile (*n* = 107)	3rd SES quintile (*n* = 145)	4th SES quintile (*n* = 167)	5th SES quintile (*n* = 183)	Linear regression B for SES percent (95% CL)		Kruskal-Wallis-Test
	Mean (SD)					adj. For age	adj. For age and health status	*p*-value
Men								
Questionnaire								
PA, hrs/week	13.73 (15.82)	11.90 (12.46)	10.46 (10.75)	7.25 (9.54)	5.44 (7.23)	**−0.23 (−0.29, −0.17)**	**−0.23 (− 0.29, − 0.17)**	**< 0.01**
MVPA, hrs/week	9.23 (11.55)	8.42 (8.33)	7.90 (7.95)	5.50 (7.32)	4.39 (5.52)	**−0.15 (− 0.19, − 0.10)**	**−0.15 (− 0.19, − 0.10)**	**< 0.01**
METs/week	56.71 (66.29)	49.23 (49.66)	46.36 (47.89)	31.77 (40.99)	25.56 (32.31)	**−0.88 (−1.14, − 0.62)**	**−0.90 (−1.16, − 0.63)**	**< 0.01**
Accelerometer								
Total PA (CPM)	1497.38 (423.85)	1449.01 (433.45)	1537.84 (412.83)	1459.62 (397.57)	1624.98 (435.61)	**3.54 (1.16, 5.92)**	**2.73 (0.31, 5.14)**	**< 0.01**

*SD* Standard deviation, *PA* Physical activity, *MVPA* Moderate to vigorous physical activity, Metabolic equivalents, *CPM* Counts per minute, *CL* Confidence limits

Statistically significant results (*p* < .05) in bold,,

Linear regressions showed significant negative associations between SES and all self-reported PA indicators for women and men. Significant positive associations were seen between objectively measured PA and SES for men (B: 2.73, 95% CL: 0.31, 5.14), but not for women (B: -0.72, 95% CL: − 3.56, 2.12).

Kruskal-Wallis-Tests showed significant differences between SES quintiles for all variables, except for objectively-measured PA in women.

Table 3 presents the time per week spent on physical activities for women and men, stratified by SES, to take a closer look at the composition of the self-reported PA variables. The results indicate that, regardless of sex, the time spent on housework, gardening, biking, and walking decreases with increasing SES. Solely exercise did not show a consistent pattern, with women in the second SES quintile and men in the third quintile reporting the most hours per week (women: 3.57 ± 4.16 h/week, men: 3.75 ± 4.60 h/week). The lowest amount of time for exercise was reported by women in the first SES quintile (2.69 ± 4.50 h/week) and men in the fifth SES quintile (3.04 ± 3.86 h/week).

**Table 3:** Time spent on self-reported physical activities by socioeconomic status stratified by sex

	Women						Men					
	1st SES quintile (*n* = 161)	2nd SES quintile (*n* = 173)	3rd SES quintile (*n* = 150)	4th SES quintile (*n* = 142)	5th SES quintile (*n* = 140)	Kruskal-Wallis-Test	1st SES quintile (*n* = 88)	2nd SES quintile (*n* = 107)	3rd SES quintile (*n* = 145)	4th SES quintile (*n* = 167)	5th SES quintile (*n* = 183)	Kruskal-Wallis-Test
	Mean (SD)					*p*-value	Mean (SD)					*p*-value
Housework, hrs/week	5.45 (7.82)	4.83 (8.61)	3.62 (6.38)	2.19 (5.31)	0.94 (2.89)	**< 0.01**	2.97 (4.31)	2.53 (4.18)	1.92 (3.67)	1.16 (2.56)	0.84 (2.33)	**< 0.01**
Gardening, hrs/week	1.80 (3.71)	1.15 (2.81)	1.07 (2.76)	0.99 (3.15)	0.27 (1.17)	**< 0.01**	3.47 (7.30)	2.35 (3.93)	1.98 (3.75)	0.93 (2.88)	0.50 (1.71)	**< 0.01**
Biking, hrs/week	1.64 (3.08)	1.44 (3.11)	1.20 (2.56)	0.75 (2.50)	0.34 (1.34)	**< 0.01**	2.25 (3.85)	1.96 (3.43)	1.65 (2.76)	1.08 (2.45)	0.73 (2.30)	**< 0.01**
Walking, hrs/week	1.69 (2.71)	1.19 (2.47)	1.04 (2.10)	0.50 (1.52)	0.21 (0.81)	**< 0.01**	1.92 (3.65)	1.55 (3.73)	1.17 (2.06)	0.74 (2.06)	0.34 (1.15)	**< 0.01**
Exercise, hrs/week	2.69 (4.50)	3.57 (4.16)	2.89 (3.56)	3.56 (5.56)	3.00 (3.05)	0.57	3.13 (5.19)	3.51 (5.29)	3.75 (4.60)	3.34 (4.82)	3.04 (3.86)	0.26

*SD* standard deviation

Statistically significant results (*p* < .05) in bold

Kruskal-Wallis-Tests showed significant differences between the SES quintiles, for women and men, for all physical activities (*p* < 0.01) except exercise (p: 0.57 for women; p: 0.26 for men).

## Discussion

In the present study, associations between SES and PA among older adults were investigated, using self-reported and objective PA measurements. Moreover, we examined the time spent on self-reported physical activities separately. Results showed decreasing mean hours per week of self-reported total PA and MVPA as well as mean METs per week with increasing SES, and significant differences between SES quintiles, regardless of sex. Objectively measured PA showed a different pattern with women in the first SES quintile having the lowest and in the third quintile the highest CPM. Men in the second SES quintile showed the lowest and in the fifth SES quintile the highest CPM. Linear regressions showed significant positive associations between objectively measured PA and SES for men, but not for women. Self-reported hours per week spent on housework, gardening as well as biking and walking for transport decreased with increasing SES, in both women and men, and were significantly different between SES quintiles. Self-reported time on exercise, however, did not show a consistent pattern.

Our results indicate that self-reported time in PA and MVPA as well as mean METs are significantly negatively associated with SES. Existing research regarding this association is contradictory. Most studies investigating the associations of PA and SES among older adults could either find no significant associations between socioeconomic factors and self-reported PA [13, 15], or they reported positive associations between education and household income with moderate and high PA, respectively [17, 35]. An Iranian study by Kazemi Karyani et al. [36] found similar results to ours, with the mean METs of self-reported activities decreasing with increasing SES. Multiple studies [37–39] reported higher levels of total PA and of walking for transport in participants with low SES, mainly through high occupational PA. Our results also showed participants in lower SES quintiles spending more time on walking and biking for transport than those in higher SES quintiles. In contrast to our study, the aforementioned studies did not solely focus on older adults, leading to higher rates of participants having an occupation. In our sample only 13.7% of women and 18.2% of men stated to have a paid occupation and a sensitivity analysis showed no differences in results (data not shown). Therefore, occupational PA is not an explanation as to why participants with lower SES showed higher levels of self-reported PA, MVPA, and METs as well as more time in active transport. A possible reason for a higher time in active transport among lower SES participants could be that lower SES groups are less likely to be able to afford a car and are more dependent on active transport modes.

In our study, participants with a lower SES spent more time doing housework and gardening than those with a higher SES. This is in line with previous research. Domestic activities are less frequently performed by older adults with a higher education [15] or higher occupational status [40]. A reason could be that a higher education often leads to higher income, resulting in being more likely to pay for help doing household chores or gardening. Regardless of SES, women tend to spend more time doing housework and men spend more time on gardening, as our previous research has shown [23].

Time spent on exercise did not show a consistent pattern regarding SES. This is in contrast to previous studies, which reported higher aerobic PA in participants with middle and high SES [14]. Higher SES groups are also more involved in moderate exercise compared to lower SES groups, which were more associated with habitual PA [41]. These findings could be explained with research showing participants with lower SES to report generally more barriers for being physically active than those with higher SES [42, 43]. Since our results do not replicate these findings, further research is needed to investigate the underlying reasons.

Objectively measured PA was positively associated with SES, which was significant in men, but not in women. A recent German study found similar results, with education being positively associated with moderate PA in older women and men, and with overall PA in men [17]. Gubelmann et al. [35], however, found contradictory results that less educated participants were more likely to be regularly active and highly educated participants concentrated their PA on weekends. Since they also included middle-aged adults and discovered a positive association between employment and high PA levels, occupational PA could be a reason for these differing results. Furthermore, they showed that higher income was associated with higher overall PA. Contrasting to our results, one study found lower SES groups spending more time in accelerometer assessed MVPA than higher SES groups [44]. The authors explained this mainly with high levels of active transport in their sample. The differing results could stem from differences in PA measurement and data analyses. In our study, accelerometers were worn on the non-dominant wrist, since it seems to have a higher compliance [45, 46] and can measure upper body movements better [5]. Gubelmann et al. [35] and Ramires et al. [44] also used wrist-placements for their studies. Kleinke et al. [17], however, placed the accelerometer on the hip.

Notably, our results showed discrepancies between self-reported and objectively measured PA. One possible explanation could be misreporting of PA in questionnaires. Research showed underreporting of MVPA among men and educated participants [47]. Dyrstad et al. [48] found sex differences in reporting MVPA with men stating higher values than women, and differences in education with lower educated participants reporting more time spent on daily PA. Misreporting could be a result of social desirability [48, 49], inadequate questionnaires [50, 51], recall bias, or decreasing cognitive function in older adults [52]. Generally, objective PA measurements, such as accelerometers, are more valid than self-reported PA [53]. Since both methods assess different aspects of PA and it is not entirely clear yet to which degree they differ in measuring PA [54], objective and self-reported PA should be seen as complementary information rather than using them interchangeably [55]. These results indicating that participants with a lower SES tend to misreport PA, are an important finding for health research and the development of PA promotion interventions regarding their focal point.

The study has some limitations that need to be addressed. Due to the cross-sectional design, conclusions regarding causation cannot be made. Longitudinal analyses need to be conducted to examine the precise associations of SES and PA among older adults. Furthermore, the questionnaire used in the OUTDOOR ACTIVE study was not a validated PA questionnaire, but rather based on existing questionnaires. This could lead to inaccurate assessments of PA. However, to date there is no unified approach on how PA among older adults should be measured, regardless of self-reported or objective measurements. These methodical differences complicate the comparisons between studies.

One strength of this study is the use of a SES index instead of using single socioeconomic dimensions. Research has shown that using only individual aspects of SES resulted in less consistent evidence regarding associations with PA compared to using an SES index [10]. Moreover, we included both self-reported and accelerometer assessed data on PA for older adults and investigated self-reported activities separately, which is scarce in existing studies, and helps to strengthen the evidence on this topic.

## Conclusion

The study found SES differences in self-reported weekly total PA and MVPA as well as mean METs per week, with participants in lower SES groups being more active. Objectively measured PA showed a different pattern with positive associations between objectively measured PA and SES, which was significant for men, but not for women. Self-reported hours per week spent on housework, gardening as well as biking and walking for transport decreased with increasing SES, in both women and men. Self-reported time on exercise, however, did not show a consistent pattern. The evidence for several determinants of PA among older adults is still insufficient. However, only few studies used objective PA measurements to assess the associations between PA and SES among older adults, which is why our results are an important contribution to strengthen the evidence on this topic. Unified approaches on measuring PA and SES of older adults, and longitudinal research are needed to understand the specific associations between the two complex constructs. This would, on the one hand, help to develop PA promotion interventions targeting specific SES groups, and on the other hand, help to develop accurate, valid, and reliable self-reported and objective measurements of PA for older adults.

## Supplementary Information

**Additional file 1.** Missing data of used variables. We have listed the number of missing data for each variable used in this study.

## Data Availability

The datasets used and/or analysed during the current study are available from the corresponding author on reasonable request.

## Abbreviations

CPM: Counts per minute
METs: Metabolic equivalents
MVPA: Moderate to vigorous physical activity
PA: Physical activity
SES: Socioeconomic status
