# Supplementary material for: Associations between socioeconomic status and physical activity among older adults: cross-sectional results from the OUTDOOR ACTIVE study
Source: BMC Geriatr. 2022 May 6;22:396. doi: 10.1186/s12877-022-03075-7 (PMC9074343; doi:10.1186/s12877-022-03075-7)
Supplement: Supplementary file 1 — Additional file 1. Missing data of used variables. We have listed the number of missing data for each variable used in this study. [file 12877_2022_3075_MOESM1_ESM.docx]

**Additional file 1** **Missing data of used variables**

| Variable | Missings |
| --- | --- |
| Marital status | 66 (4.4%) |
| Socioeconomic status | 51 (3.4%) |
| Occupational status | 74 (4.9%) |
| Self-reported health | 60 (4.0%) |
| Age | 0 |
| All self-reported physical activity | 57 (3.8%) |
| Total physical activity (CPM) | 0 |
| CPM: Counts per minute |  |
